# Arl3 regulates a transport system for farnesylated cargo

**DOI:** 10.1186/2046-2530-1-S1-P71

**Published:** 2012-11-16

**Authors:** S Ismail, YC Chen, AR Rusinnova, AC Chandra, MB Bierbaum, LG Gremer, GT Triola, HW Waldmann, PB Batiaens, AW Winttinghofer

**Affiliations:** 1 Max Planck Institute for Molecular Physiology, Germany

## 

Arl3 is a small G-protein that is found exclusively in ciliated organisms. In addition, knocking out of Arl3 results in a plethora of ciliopathies. Arl3 is known to bind the photoreceptor (specialized cilia) specific PDE delta subunit (PDE6D), which in turn bind to prenylated proteins. The significance of this interaction and the function of Arl3 in cilia are poorly understood. Here in this study, by solving the crystal structure of a fully modified prenylated (farnesylated) Rheb in complex with PDE6D and comparing it to a structure of PDE6D in complex with the Arl3 homologue Arl2, we show that Arl3 is an allosteric regulator of PDE6D. Arl3, in a nucleotide dependent manner, releases the farnesylated cargo bound to PDE6D. We explain the molecular mechanism of this release and we further verify the mechanism in vitro and by live cell imaging. Based on this study we hypothesize that Arl3 regulate the targeting of prenylated cargo in and out the cilia.

